# Efficacy and safety of intradermal vaccination against porcine circovirus type 2 and *Mycoplasma hyopneumoniae* under combined exposure field conditions

**DOI:** 10.1186/s40813-025-00431-y

**Published:** 2025-04-07

**Authors:** Panagiotis Tassis, Suzanne Pel, Dimitrios Floros, Kim ter Haar, Qi Cao, Ioannis Tsakmakidis, Vassileios Papatsiros, Niki Ntarampa, Ioannis Arsenakis, Eleni D. Tzika

**Affiliations:** 1 Farm Animals Clinic, School of Veterinary Medicine, Aristotle University of Thessaloniki, Thessaloniki, Greece; 2 MSD Animal Health, Boxmeer, The Netherlands; 3 Clinic of Medicine, School of Veterinary Medicine, University of Thessaly, Karditsa, Greece; 4 Laboratory of Anatomy & Physiology of Farm Animals, Agricultural University of Athens, Athens, Greece

**Keywords:** Pigs, Intradermal vaccination, Porcine circovirus type 2, *Mycoplasma hyopneumoniae*, Lung lesions, Pleurisy, Viraemia, Enzootic pneumonia, Porcine respiratory disease complex

## Abstract

**Background:**

Porcine circovirus 2 (PCV2) and *Mycoplasma hyopneumoniae* (M hyo) are two of the most important swine pathogens with variable clinical presence in swine farms globally, affecting health and performance of pigs under field conditions. The primary objective of this study was to assess the efficacy of a ready to use intradermal (ID) vaccine (Porcilis PCV M Hyo ID, MSD Animal Health, The Netherlands) against PCV2 associated disease and M hyo associated enzootic pneumonia under practical (field) conditions. In addition, the safety of the test product was clinically assessed, as the study animals were examined for general and local side effects after vaccination. A total of 678 animals in a Greek farrow to finish farm were equally divided in two trial groups (test and control group). Test group animals received the test vaccine at the age of three weeks, while control group animals remained unvaccinated. Parameters regarding health [lung lesion score (LLS) and pleurisy scoring, PCV2 viraemia and shedding] and performance [body weight (BW), average daily weight gain (ADWG)] were recorded and evaluated.

**Results:**

Vaccination improved the ADWG during both the finishing period (improvement by 34 g; *p* < 0.0001), and the overall period (increase by 24 g; *p* < 0.0001). Moreover, reduced mean LLS values (*p* < 0.0001), as well as reduced percentage of animals with pleurisy (*p* = 0.0082) and a decrease in PCV2 viraemia (*p* < 0.0001) and viral shedding (*p* = 0.0181) were observed in vaccinated animals when compared with the unvaccinated controls. As regards safety, a slight local skin reaction at the site of vaccination was detected and in two pigs a mild systemic reaction was reported with full recovery.

**Conclusions:**

Findings suggested that the test vaccine is safe and effective against both PCV2 and M hyo associated diseases in vivo, thus it’s use as part of a vaccination programme under field conditions is expected to improve respective health and performance parameters in pigs.

## Background

Porcine circovirus 2 (PCV2) and *Mycoplasma hyopneumoniae* (M hyo) are among the most important swine pathogens. They can induce respiratory disorders in swine, thus resulting in negative financial balance of swine production. Both agents are part of the etiology of the porcine respiratory disease complex (PRDC) [1]. The cell wall-free bacterium M hyo is the aetiological agent of enzootic pneumonia (EP) as a chronic lung infection which impacts growth and is characterized by significant morbidity and low mortality [2]. It has been reported that more than 70% of swine herds globally are infected with M hyo [3]. Diagnosis of M hyo infections on farms are mainly performed in growers and finisher pigs. M hyo is characterized as a “slow bacterium” in terms of infection dynamics with low transmission rate whereas clinical signs become apparent only after weeks of infection and recovery can be lengthy [4–6].

PCV2 is a non-enveloped circular single stranded DNA virus belonging to the Circoviridae family and is responsible for the induction of several pathologies suggested as porcine circovirus diseases (PCVD), such as subclinical infection (PCV-2-SI), systemic (PCV-2-SD) and reproductive (PCV-2-RD) diseases, and porcine dermatitis and nephropathy syndrome (PDNS) [7]. PCV2-SD’s clinical picture includes wasting, decreased weight gain, ill thrift or poor-doing animals, sometimes with respiratory and/or digestive disorders, whereas PCV-2-SI is observed with decreased average daily weight gain (approx. 10–40 g/day) without any evident clinical signs [7, 8]. Studies have demonstrated the global presence of concomitant M hyo and PCV2 infections in swine farms [9], as well as their significant impact on health and performance of swine [10], whereas a synergistic effect of both infections has also been reported in experimental models [11, 12].

Vaccination programmes including vaccines against both PCV2 and M hyo have a significant preventive role against respective diseases on farms. Previous studies have reported the efficacy of mono- or bivalent intramuscular (IM) vaccines against one or both pathogens [12, 13]. Quite similarly to the IM route, the intradermal vaccination (ID) vaccination with mono- or bivalent vaccines provided beneficial results in terms of health and performance of swine against various pathogens under field conditions [14–17]. Major benefits observed in animals vaccinated against PCV2 and M hyo, either through IM or ID route, include the reduction of the prevalence of broncho pneumonic lungs and those affected by cranioventral pleurisy, as well as the reduction of EP-like lung lesions and PCV2-viremia, improved antibody levels against both pathogens, increased average daily weight gain (ADWG) and decrease of the rate of antibiotically treated animals against respiratory disease [13, 18–20]. The mode of action of ID vaccination relies on the stimulation of a protective immune response through antigen-presenting cells in the dermis, near skin-draining lymph nodes [21, 22]. Significant welfare benefits have been reported for ID vaccine administration, regarding the reduction of pain and stress associated with IM vaccination [23, 24]. In addition, the biosecurity risks of pathogens transmission with the use of needles and the “needle breakage in the pig” risk of IM vaccination are not observed with the use of ID vaccination. User convenience of one-shot vaccination with automated and easy to handle needleless devices which provide information to the user (e.g. number of shots given per vial) and connectivity should be also highlighted as beneficial for implementation under field conditions [25].

The long-term implementation of vaccination strategies against PCV2 and M hyo globally has resulted in observing fewer acute cases of PCVD and EP, but greater subclinical circulation of the pathogens under field conditions [26–27]. In contrast, PCV2 or M hyo concomitant infections along with other viral or bacterial pathogens seem to be more frequently observed [28]. A bacterium-virus or a virus-bacterium coinfection of the respiratory tract in swine frequently results in an aggravation of pulmonary lesions with greater inflammatory and immune response [1]. The economic impacts of co-existing pathogens involved in the PRDC, and the costs and benefits of interventions such as vaccination programmes have been demonstrated by Boeters et al. [29], interpreting data from studies conducted in 23 countries. Findings suggested reduced medication cost per pig, reduced labour costs and increased annual profit in farms due to different vaccination schemes against M hyo, whereas Kaalberg et al. [30] reported that vaccination strategies against PCV2 provided return on investment per pig and significant benefit of 2.84 € per finisher.

Though research data on monovalent and bivalent IM vaccinations against PCV2 and M hyo are increasing in the last decades, research data on the efficacy of ID bivalent vaccination against both pathogens are still scarce. Therefore, the aim of the present study was to evaluate the efficacy of a novel ready to use combined ID vaccine against both PCV2 and M hyo under field conditions with both pathogens circulating in the farm. In addition, the study animals were observed for side effects, to evaluate the safety of the test ID vaccine.

## Methods

### Farm and animals

The study farm is a 660-sow farrow-to-finish unit with its own feed-mill and slaughterhouse. It has a weekly production system and applies an all-in all-out management system. After weaning, at 28 days of age, the piglets were sorted by weight and moved to flat decks in the nursery unit until 65 days of age. From the nursery, the piglets are moved to the grower unit (standard pens with slatted floors) and about 9 weeks later to the finishing unit. Slaughter age varies between 160 and 200 days of age.

Pretrial evaluation of lung lesions from the selected farm (132 lungs inspected) according to the method described in Ph. Eur. Monograph 2448 “Porcine enzootic pneumonia vaccine inactivated”, provided evidence of an M hyo infected farm (lesion score of 13.7 prior to the study), whereas seropositivity for M hyo was present in samples from the age of 10 weeks up to slaughter age. Laboratory results also indicated antibody response to PCV2 in all age groups (i.e. animals at 3, 5, 10, 15 and 20 weeks of age, as well as in samples from gilts and sows) and PCR detection of the virus slightly increasing in pigs at 10 weeks of age (data not presented).

### Test product

The test vaccine (Porcilis PCV M Hyo ID; MSD Animal Health, The Netherlands) is a newly developed inactivated vaccine for ID administration against PCV2 and M hyo in pigs. The vaccine has already shown to be safe and efficacious under controlled (laboratory) conditions (data not presented). It contains Baculovirus expressed PCV2b ORF2 subunits (inactivated) and inactivated M hyo type J with a synthetic squalane-based adjuvant.

### Clinical procedures and parameters

The study was carried out according to a controlled, randomized, and masked design in a Greek pig farm with a confirmed M hyo and PCV2 infection. In total 678 healthy suckling piglets were selected at three weeks of age (19–21 days) from two production batches and randomly allocated to one of two equally sized groups. The animals were allocated according to a randomization list per production batch to one of the two treatment groups until the required number of piglets was reached. This randomization list was generated before the start of the study with the software nQuery [31]. The animals of the test group (338 pigs) received the test ID vaccine at three weeks of age, whereas the control group animals (340 pigs) were not vaccinated. ID administration of the test product at the dose of 0.2 ml was performed with the use of IDAL^®^ 3G injector (Henke SASS Wolf GmbH, Tuttlingen, Germany). No other vaccine was administered to the test animals through the course of the study. Male and female animals introduced in the study were balanced and all animals were ear tagged at vaccination. Feed and water were available *ad libitum*. Piglets from the two treatment groups were commingled and housed together.

To assess the efficacy against M hyo, the M hyo associated lung lesion scores (LLS) at slaughter and the ADWG in the finishing period (from 9 weeks of age up to slaughter at 23 weeks of age) were set as primary efficacy parameters. Respective primary parameters for the assessment of the test product’s efficacy against PCV2, were the PCV2 viraemia (qPCR viral load) and the ADWG evaluation. Secondary efficacy variables were the overall ADWG (from vaccination to slaughter), mortality, morbidity (defined as the proportion of animals requiring individual treatment, irrespective of the number of individual treatments), pleurisy lesions, and PCV2 faecal shedding. In addition to the efficacy parameters, the serological response to vaccination was also determined.

Body weight (BW) of the study pigs was assessed individually at admission, at transfer to the grower unit (9 weeks of age), and just before slaughter (23 weeks of age). The lungs of all animals from the two groups were examined individually at slaughter by a pathologist (blinded to the trial groups) to score the severity of lung lesions and pleurisy. Group medication could only be given when necessary, according to respective veterinary diagnosis, and it was recorded, whereas pigs that died during the study were examined to establish the cause of death.

One piglet per treatment group from 30 litters, was selected for collection of a rectal swab and blood sampling. The blood samples and rectal swabs were collected at regular intervals during the study (i.e., on day 0 and on week 4 blood only, and on weeks 7, 10, 13, 16, 19 of the study both blood and rectal swab samples), for determination of the PCV2 quantity by qPCR [31]. Vials with medium (Sigma Virocult^®^ type 30MW950S) were used for rectal swabs (Medical Wire & Equipment, Corsham, Wiltshire, England), whereas BD vacutainer tubes (BD-Plymouth, UK) were used for blood sampling. Blood samples were refrigerated overnight prior to centrifuging at 3,000 rpm X 10 min. for serum collection. Serum was stored at -20^o^C until analysis.

### Serum analysis

The sera were analyzed by M hyo ELISA (Swine HerdCheck M Hyo IDEXX) as per manufacturer instructions and PCV2 ELISA (PCV2 ELISA MSD Animal Health). For the PCV2 ELISA, serially diluted serum samples were incubated on microtiter plates coated with baculovirus expressed PCV2 Orf2 antigen. After removing the serum, all wells were incubated with a fixed amount of biotin labelled PCV2-specific monoclonal antibody. Next bound MoAb was incubated with peroxidase conjugated avidin followed by chromophoric detection. Titres were expressed as the reciprocal of the serum dilution with a calculated extinction value of 50% maximum extinction. The results were expressed as log2 titres. For calculation of the mean PCV2 antibody titres the values outside the detection limits were set one dilution step lower or higher: i.e., > 16 was set at 17 and < 2 was set at 1.

The last set of samples before slaughter (19 weeks post vaccination) was tested for antibodies against co-infections. The tests for LI (*Lawsonia intracellularis*) ELISA MSD Animal Health, APP (*Actinobacillus pleuropneumoniae*) ELISA (OMP) MSD Animal Health, HPS (*Haemophilus parasuis*– *Glässerella parasuis*) Biochek, PRRSV (Porcine Reproductive and Respiratory syndrome virus) ELISA (IDEXX), Swine Influenza hemagglutination inhibition test for types H1N1 (EA & pdm), H1N2 (ScotCL2), and H3N2 (gent84) were performed according to manufacturer’s instructions.

### Lung lesion scores

LLS were evaluated with the same method as in the pre-trial investigation (Ph. Eur. Monograph 2448 “Porcine enzootic pneumonia vaccine inactivated”). For each lung lobe, the percentage of the surface (0-100%) with signs of a M hyo infection associated inflammation (consolidated, grey to purple colored) was estimated and recorded. These percentages (in fact proportions) were multiplied with a respective weighing factor and added to obtain the total LLS. Weighing factors were: 5 and 11 for the right and left cranial lobe respectively, 6 and 10 for the right and left medial lobe respectively, 29 and 34 for the right and left caudal lobe respectively, whereas a weighing factor of 5 was set for the accessory lobe. Thus, the minimum LLS for each animal was 0 and the maximum 100. Moreover, the lungs were scored for the presence of pleuritis lesions as follows: 0 = absent, 1 = topical adhesions (spots) and 2 = larger adhesions (larger than spots in one or more lobes).

### PCV2 molecular analysis

Briefly, DNA was isolated from swabs or sera. The amount of PCV2 (ORF2) genomic DNA was quantified by PCR [32]. All samples were included in duplicate per extraction run and the results were expressed as log10 copies/µl DNA extract. The mean of both duplicate samples was calculated. A PCR reaction was considered positive (i.e., above the limit of quantification) if the mean copy number of the samples was ≥ 1.00 log10 c/µl DNA. Results below 1.00 log10 c/µl DNA were reported as 0.00 log10 c/µl DNA. Similarly, all rectal swabs were tested by qPCR for the detection of PCV2 virus shedding as described above [32].

### Safety assessment

Side effects that occurred during or after vaccination were recorded. Briefly, all study animals were observed during and immediately after vaccination for the detection of general immediate reactions such as tremors or convulsions and 60 selected animals (two animals from 30 litters, equally divided per trial group) were observed individually for local reactions (type and size) and systemic reactions on day 0 and 7, 14, 21 and 28 days after vaccination.

### Statistical analysis

The statistical analysis was performed by means of the software package SAS^®^ (version 9.4, SAS Institute Inc., Cary, NC, USA). The individual pig was the experimental unit. Sample size calculation of the study resulted in at least 300 pigs per group with 80% power to detect a mean ADWG difference of 25 g among trial groups, assuming that the common standard deviation was 100 g using a two-group t-test with a 0.05 two-sided significance level. Sample size of at least 300 pigs / group had a power of 80% to detect a difference of 0.15 in log10 transformed LLS assuming that the common standard deviation was 0.6 in a parallel two group study design with a two-sided significance level of 0.05. For PCV2 molecular analysis regarding viraemia and shedding parameters, a sample size of at least 25 animals in each group had 80% power to detect a probability of 0.750 that an observation in the vaccinated group was less than an observation in the control group using a Wilcoxon (Mann-Whitney) rank-sum test with a 0.05 two-sided significance level.

The ADWG and the log-transformed LLS were compared between groups using mixed model ANOVA. In the log-transformed LLS analysis, the treatment was included as the fixed effect. The occurrence of lungs with LLS greater than 0 was analyzed using Fisher’s exact test. In the ADWG analysis, initial BW was included in the model as a covariate, whereas treatment and gender with appropriate interactions were included as fixed effects and production batch and sow as random effects. The AUC (Area Under the Curve) of the PCV2 viraemia and shedding data were calculated in terms of the Trapezoidal rule and analysed using a mixed effect ANOVA model with treatment as the fixed effect and production batch as the random effect. The incidence of viremic animals was analyzed using the Cochran Mantel Haenszel (CMH) method with production batch as the classification variable. The duration of viraemia was analyzed using a mixed model ANOVA with treatment as the fixed effect and production batch as the random effect. Mortality, morbidity, and pleurisy observed were compared between groups using the CMH method with production batch as the classification variable. In the inferential statistical analyses of the efficacy variables, the tests were two sided and 0.05 was the significance level. Descriptive statistics was used to summarize and present safety observations results.

## Results

Out of the 678 animals included in the study, 615 animals were evaluated at slaughter due to loss of ear tags (40 animals) or mortality/culling reasons (23 animals).

### Performance parameters, mortality and morbidity

**Fig. 1:**
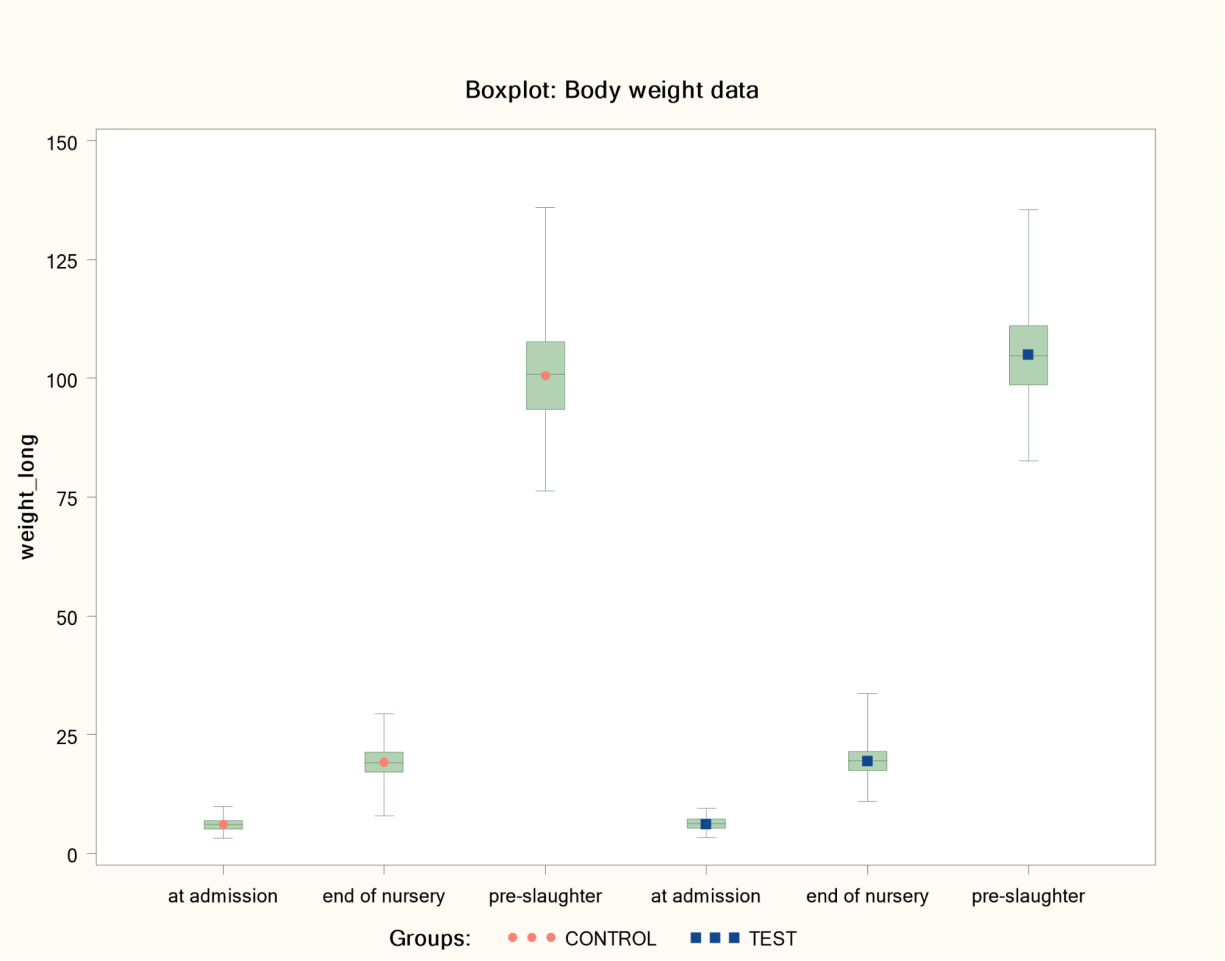
Boxplot of body weight (kg) per treatment group^∋^ in each trial stage. ^∋^ Control group: Unvaccinated animals; Test group: Animals vaccinated with Porcilis PCV M Hyo ID at three weeks of age

The BW data indicated that pre-slaughter control pigs had a wider range of weights in comparison to the test group. Range of BW at slaughter in the control group was between 76.2 and 135.9 kg, whereas BW range in the test group was between 82.6 and 135.5 kg. Moreover, the mean ADWG values in the test group were significantly improved during both, finishing and the overall trial period (mixed model ANOVA: *p* < 0.0001). Table 1 presents results of BW and ADWG by treatment group and study period, whereas Fig. 1 demonstrates a boxplot of BW alterations among groups in the time phases of the trial.

**Table 1:** Body weight (BW) and average daily weight gain (ADWG) values per trial group and period

	BW (kg) [mean ± standard deviation (SD)]			
Treatment group^#^	BW day 0	BW end of nursery	BW at slaughter	
Control group	6.0 ± 1.30	19.2 ± 3.36	100.5 ± 9.98	
Test group	6.3 ± 1.29	19.5 ± 3.30	105.1 ± 8.65	

	ADWG (g) [mean ± standard error of the means (SEM)]			
	Nursery period	Finishing period	Total study period	
Control group	325.5 ± 24.3^a^	795.2 ± 13.2^a^	661.4 ± 7.4^a^	
Test group	323.1 ± 24.3^a^	829.5 ± 13.0^b^	685.9 ± 7.2^b^	

^a, b^ Means with different superscripts in the same column differ significantly (*p* < 0.0001 in both ADWG comparisons at the finishing and the total trial period)

^#^ Control group: Unvaccinated animals; Test group: Animals vaccinated with Porcilis PCV M Hyo ID at three weeks of age

As regards mortality, 23 pigs (3.4%) died or were culled during the study period in total; 14 out of 340 animals (4.1%) were from the control group and 9 out of 338 (2.7%) were from the test group. Gross pathology evaluation suggested that findings in 12 out of 23 cases (9 from the control and 3 from the treatment group, respectively) could be attributed to pathogens involved in PRDC aetiology (i.e. presence of EP-like lesions and interstitial pneumonia along with enlarged tracheobronchial lymph nodes). The mortality rate was not significantly different between the treatment groups (CMH test: *p* = 0.2957). Evaluation of morbidity, as individual medication administered to trial animals, demonstrated that 41 pigs received individual medication in total, 26 for gastro-intestinal problems and 15 for respiratory disease (mainly after the 12th week of the study). Twenty-five animals (7.4%) were from the control group and 16 (4.7%) were from the test group, suggesting the absence of a significant difference between trial groups (CMH test: *p* = 0.1519).

### Lung lesion scores

In total 615 lungs were scored. The LLS of the vaccinated animals were significantly lower compared to those of the control group (mixed model ANOVA: *p* < 0.0001; median scores of 3.2 and 6.5 in the test and control group, respectively). Lung lesions were observed in 400 lungs. A significant difference (Fisher’s exact test: *p* = 0.0002) was observed in the presence of lungs scored with LLS > 0 among groups. The 224 cases out of 310 lungs scored with LLS > 0 (72%) were observed in animals of the control group, and 176 out of 305 (58%) were from the test group. As regards pleuritis findings, 614 lungs were scored and a total of 181 (29%) lungs from both trial groups had pleuritis. On the other hand, 433 lungs didn’t have pleuritis findings, of which 203 (47%) were from the control group, and 230 (53%) were from the test group. A significant difference (CMH test: *p* = 0.0082) in pleuritis findings among groups was observed. Table 2 presents results of LLS and pleuritis findings.

**Table 2:** Lung lesion scores (LLS) and pleuritis scores per trial group

	LLS^∗^ and pleuritis findings^∋^			
Treatment group^#^	LLS	Absence of pleuritis findings	Topical adhesions (spots)	Larger adhesions
Control group	9.60 ± 11.05^a^	203 (65.7%)	70 (22.7%)	36 (11.7%)
Test group	4.31 ± 6.90^b^	230 (75.4%)	50 (16.4%)	25 (8.2%)

^∗^ mean ± standard deviation (SD)

^a, b^ Means with different superscripts in the same column differ significantly (*p* < 0.0001)

^∋^Number of cases in each category [% of total cases (309 total cases in control and 305 total cases in trial group, respectively) scored]. Comparison of pleuritis findings among groups: *p* = 0.0082

^#^ Control group: Unvaccinated animals; Test group: Animals vaccinated with Porcilis PCV M Hyo ID at three weeks of age

### PCV2 viraemia, shedding, and sequencing

**Fig. 2:**
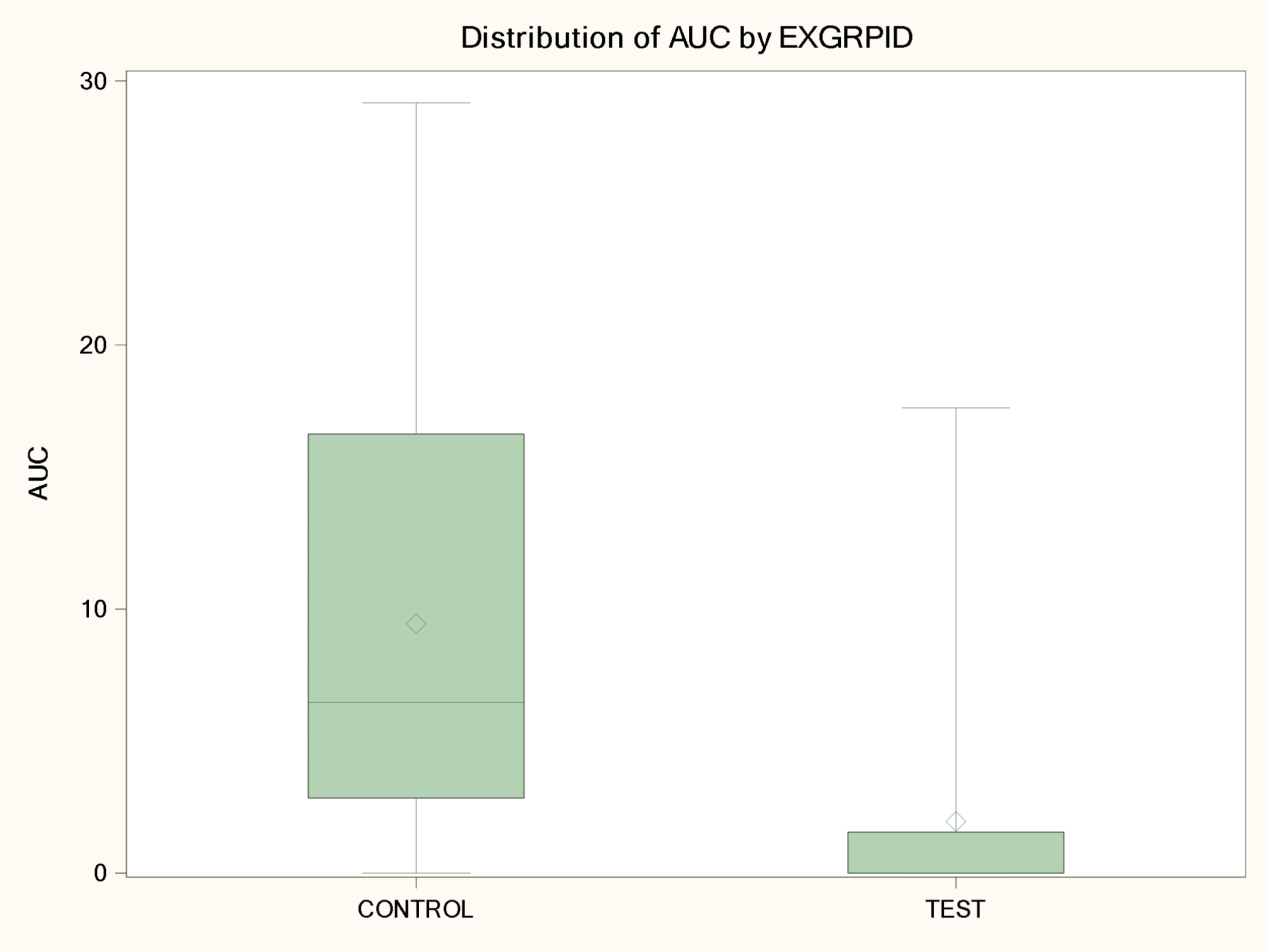
Boxplot of PCV2 viremia area under the curve (AUC) per treatment group^∋^. ^∋^ Control group: Unvaccinated animals; Test group: Animals vaccinated with Porcilis PCV M Hyo ID at three weeks of age. PCV2: porcine circovirus

**Fig. 3:**
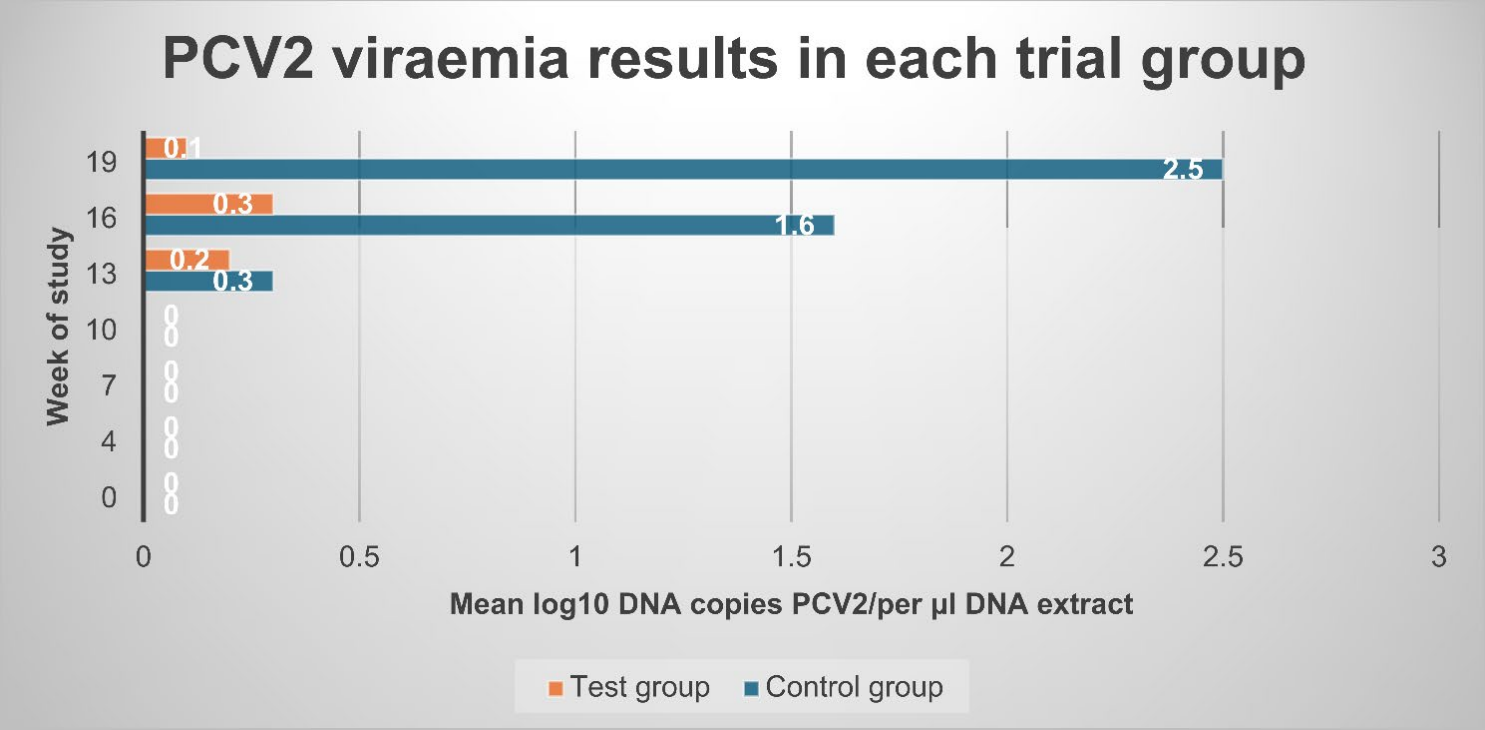
PCV2 viraemia results in each trial group at each sampling point during the study^∋^. ^∋^ Control group: Unvaccinated animals; Test group: Animals vaccinated with Porcilis PCV M Hyo ID at three weeks of age. PCV2: porcine circovirus

PCV2 viremia was observed from the 13th week of the study onwards. PCV2 viraemia as determined by mean AUC was significantly reduced (mixed model ANOVA: *p* < 0.0001) in the test group (mean AUC of 2.0) when compared to the control group (mean AUC of 9.4). A significant difference (CMH test: *p* = 0.0007) among viremic animals in each group was detected, since only six out of 30 (20%) test group animals became viremic, in comparison with 23 out of 30 control animals (77%) with viraemia. Viraemia lasted approximately 0.9 weeks on average in the test group animals, whereas a significantly longer duration of 3.7 weeks was observed in the control group (mixed model ANOVA: *p* < 0.0001). Figure 2 demonstrates graphically the PCV2 viraemia observations among trial groups, whereas Fig. 3 provides data of PCV2 viremia in each trial group / sampling time point.

**Fig. 4:**
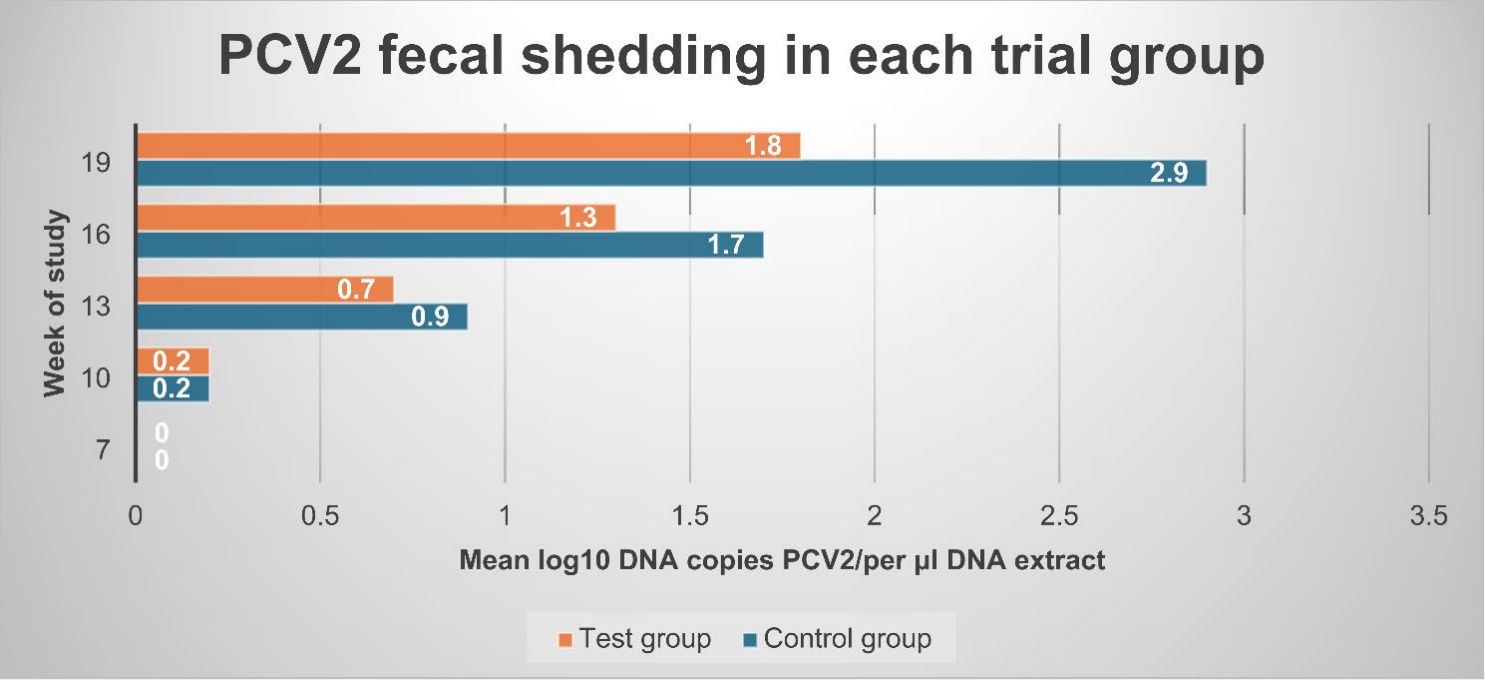
PCV2 fecal shedding results in each trial group at each sampling point during the study^∋^. ^∋^ Control group: Unvaccinated animals; Test group: Animals vaccinated with Porcilis PCV M Hyo ID at three weeks of age. PCV2: porcine circovirus

Findings of PCV2 shedding according to qPCR in rectal swabs were observed from the 10th week of the study onwards. Results provided evidence of significantly lower (mixed model ANOVA: *p* = 0.0181) shedding in the test group (mean AUC of 8.3) when compared with the control group (mean AUC of 12.4). On the other hand, PCV2 full genome sequencing results showed that PCV type 2d was circulating at the study farm during the study (data not presented). Figure 4 provides data of PCV2 fecal shedding in each trial group during the study.

### Antibody response and evidence of co-infections

**Fig. 5:**
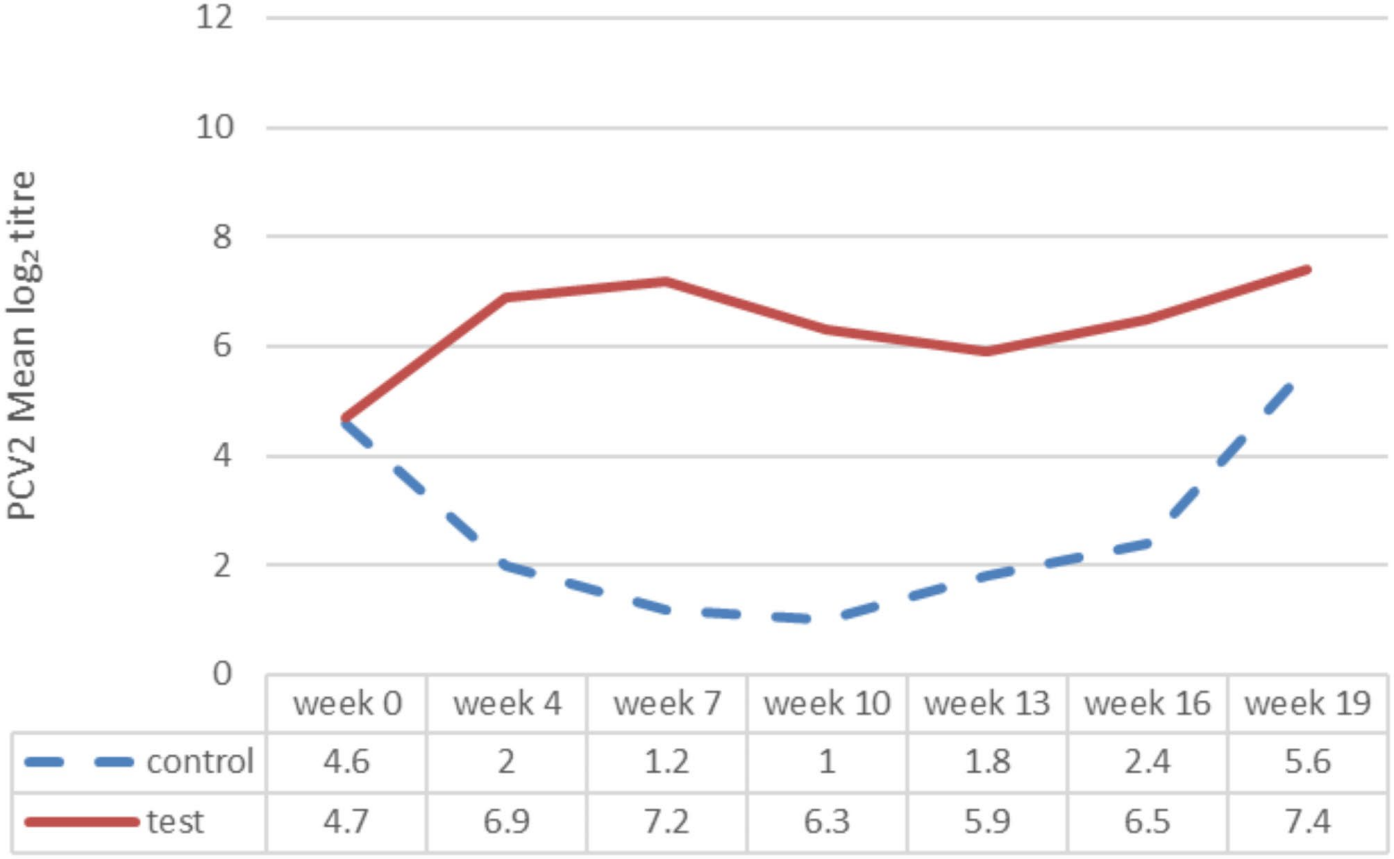
PCV2 mean log₂ antibody titre by treatment group^∋^ and week of the study. ^∋^ Control group: Unvaccinated animals; Test group: Animals vaccinated with Porcilis PCV M Hyo ID at three weeks of age. PCV2: porcine circovirus

On day 0 of the study, test group animals had a mean PCV2 antibody titre of 4.7, which was similar to that of control animals (mean antibody titre of 4.6). Results at weeks 4, 7 and 10 demonstrated a decrease of titers in the control group whereas a seroconversion due to vaccination was present in the test group animals at the same time. From week 13 onward, the mean antibody titres of control pigs started to increase and at weeks 16 and 19 titers in both groups seemed to have a parallel increasing development, suggesting that a natural PCV2 infection occurred at the beginning of the growing period. Alterations of mean PCV2 antibody titres through the course of the study are showed in Fig. 5.

**Fig. 6:**
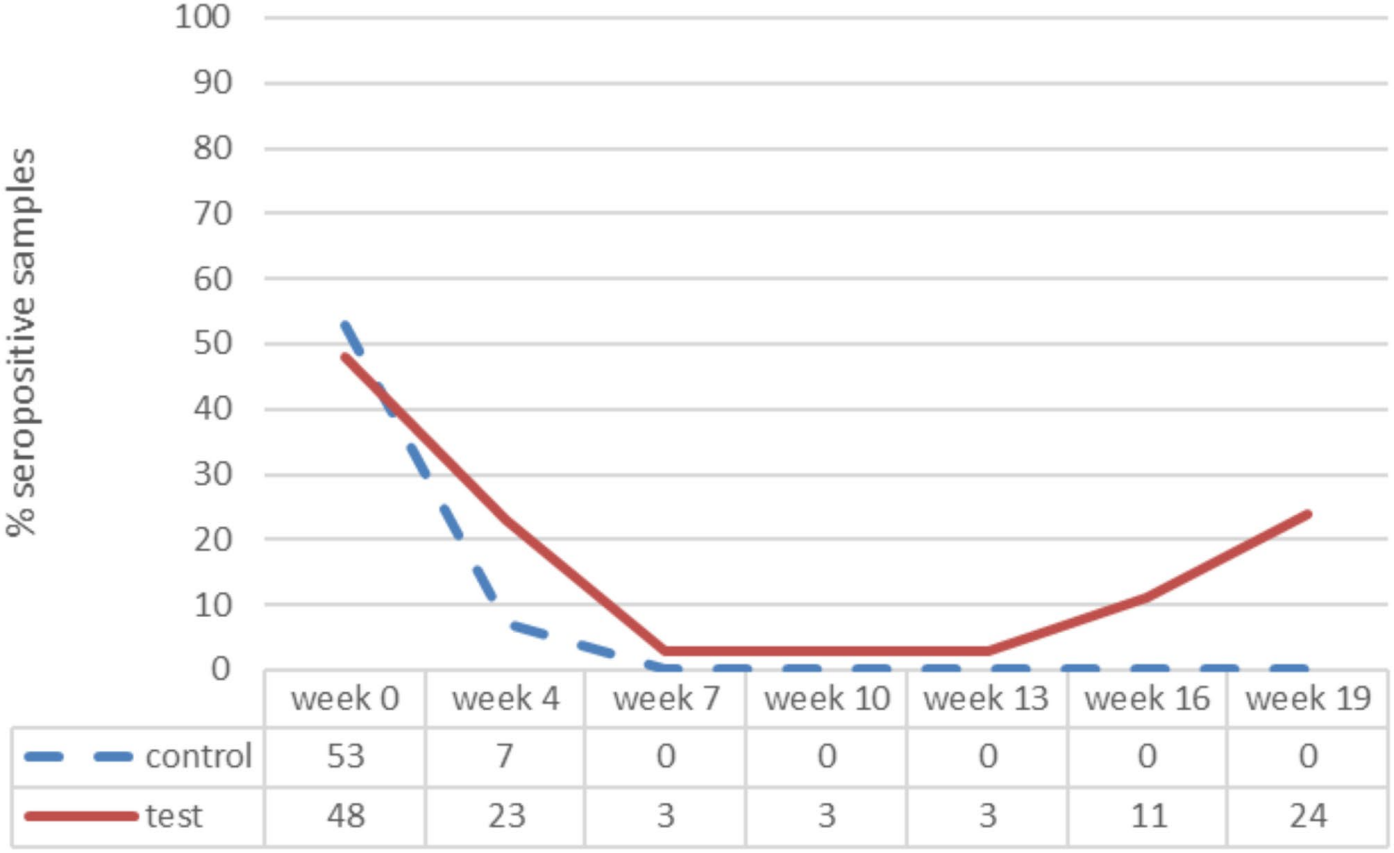
Mhyo % seropositive samples by treatment group^∋^ and week of the study. ^∋^ Control group: Unvaccinated animals; Test group: Animals vaccinated with Porcilis PCV M Hyo ID at three weeks of age. Mhyo: Mycoplasma hyopneumoniae

Figure 6 presents the variation of M hyo antibody titres between groups and time points of the study. Approximately half of the piglets from both groups had antibodies against M hyo on day 0 of the study, whereas at the end of the study antibodies were detected in 24% of the vaccinated pigs, while none of the control animals showed any presence of antibodies against M hyo. Such findings represent the immune reaction to M hyo field infection at the trial farm. Quite similarly to PCV2, a M hyo field infection should have occurred around 10–12 weeks of age, since titers increased only in the vaccinated group after the 13th week of the study.

As regards findings of simultaneous presence of other pathogens in the trial animals during the study, serological evaluation on week 19 of the study suggested absence of marked differences in antibody titers against concurrent viral or bacterial pathogens among trial groups (data not shown). Therefore, the presence of an acute co-infection of trial animals at that time point cannot be reported.

### Safety observations

Regarding immediate reactions after vaccination, one animal reacted with a transient swelling around the lower surface of the neck and swelling around the eyes. Upon checking on day 7 this animal was scored as healthy. One other test pig tended to lie down with minor signs of discomfort at day 14 after vaccination but recovered when checked on day 21. The timing makes it very unlikely that this adverse reaction was caused by vaccination. No other systemic adverse reactions were observed in vaccinated animals.

The assessment of local reactions over the four weeks following vaccination revealed that all vaccinated animals exhibited such reactions, while none were observed in the control pigs. Vaccinated pigs had mild local reactions at the site of injection with a maximum measured diameter of 5 cm. Reactions which remained on day 28 (*n* = 18) had a reduced diameter of 0.2–2.5 cm.

## Discussion

The results of the present study demonstrated that vaccination with the test ID vaccine (Porcilis PCV M Hyo ID) significantly reduced the severity of lung lesions associated with M hyo infection and pleuritic findings, as well as PCV2 viraemia and shedding. Moreover, improved performance parameters in vaccinated animals such as greater ADWG in the finishing and the overall study period in comparison with the control group, were detected. Furthermore, increased BW at slaughter with a mean improvement of 4.6 kg BW in vaccinated pigs was noticed along with improved uniformity of the test group animals’ BW at slaughter. As regards the reduction of LLS, 42% of assessed lungs from the test group showed absence of lung lesions, whereas only 28% of lungs from control animals were “free” from lung lesions.

A recent study with another one-dose ready to use combined PCV2 and M hyo ID vaccine (administration on the 24th day of age) provided also evidence of a decrease in LLS and overall prevalence of broncho pneumonic lungs [25]. In the latter study, results on vaccine efficacy related to PCV2 viremia and PCV2 shedding were not assessed, thus not presented. In another study by Suh et al. [33], findings from vaccination of 21 day old piglets with the above-mentioned bivalent ID vaccine [25] and a following experimental infection either with M hyo, or PCV2d or both pathogens, provided evidence of improved growth performance, reduced pulmonary and lymphoid lesions, increased neutralizing antibodies against PCV2d and specific interferon-γ secreting cells for each pathogen, along with reduced viral load in the blood, and M hyo load in the larynx. Results of our study on the other hand, demonstrated the immunological reaction and the improvement of primary efficacy parameters in vaccinated animals even in field conditions without the acute infection dynamics and pressure of experimental infection conditions. A significant reduction of viral shedding and a greater immune response against PCV2 natural infection at around 12 weeks of age was observed in vaccinated animals when compared with controls in the present study. At a similar timepoint, when natural exposure to M hyo antigenic load occurred in the trial farm, the immune response of vaccinated animals showed a timely response to the pathogen providing clinical protection to the animals at the last part of the study. Thus, ID vaccination with the test product in our study was able to provide significant and timely progressed immune response, counteracting antigenic pressure by both pathogens under field conditions. On the other hand, the reduced sensitivity of the ELISA test used in the study (approximately 55.69%) as regards the detection of a possibly weak immune response of control animals after field infection, could be also considered as responsible for the absence of seroconversion against M hyo in non-vaccinated animals [34]. Moreover, M hyo is not invasive and attaches to the ciliated respiratory epithelium, whereas potential colonization of the lower respiratory tract does not always guarantee an infection sufficient to trigger a humoral immune response [35]. Therefore, the serum antibody response to the bacteria may be variable [36].

Protection against both M hyo and PCV2 circulating in the trial farm, as observed with reduced pneumonia findings and viral shedding in the present study, helps to mitigate the risk of PRDC, since both pathogens have a significant role in the pathogenesis of PRDC [37]. Such protection against major PRDC aetiological factors along with the improved BW and ADWG values [4.6 kg greater weight at slaughter and 24.5 g improvement of ADWG for the total study period (*p* < 0.0001)] in vaccinated animals when compared with control animals, could be also translated as reduction of production losses and possible improvement of the financial outcome of pig production. According to the review by Boeters et al. [29], the median economic impact of single or combined respiratory infections could range from €1.70 per nursery pig up to €323 per sow per year. Improvement of such financial figures, including the reduction of antimicrobials usage due to proper preventive vaccination strategies [38], should benefit the pig industry which already works under very tight financial margins.

Evaluation of BW at slaughter and the narrower range of BW values in the test group was an indication that the mean BW of vaccinated animals at slaughter was more homogeneous than the control group. The homogeneity of weight at slaughter is always preferable in pig production, as significant financial consequences of a greater BW variability at slaughter are related to quality classification and quotation of the carcasses, whereas undoubtedly lightest pigs within a batch, will be depreciated [39–40]. Though quality classification at slaughter was not performed in the present study, our findings support a better uniformity of the final product, which could subsequently meet quality specifications and better satisfy processing requirements, as meat retailers request a more uniform product [41] and consumers demand improved product quality [42].

The dermis is an exceptional site for vaccine delivery and rich in dendritic cells and lymph vessels which promote processing of incoming antigens [43]. Findings from a previous study by Puig et al. [44] with a bivalent ID vaccine against PCV2 and M hyo (vaccination of animals on the 21st day of age), demonstrated also improved growth performance during the first weeks after weaning in comparison with groups of animals which received monovalent IM vaccines against each of the pathogens at the same timepoint. The above-mentioned findings of studies with bivalent ID vaccines against PCV2 and M hyo, support future vaccination strategic planning against both pathogens with one ID intervention, based on the infection status of the farm. It could be expected that, when serologically justified, ID vaccination with bivalent or trivalent vaccines will replace the traditional IM vaccination with monovalent vaccines. Furthermore, as observed in the present study only mild local reactions should be expected after proper ID vaccination in pigs, thus providing a safe alternative to IM vaccination. Therefore, disease prevention based on least invasive ID vaccination strategies causing minimal distress to animals with one instead of two separate restraint and vaccination efforts seems to be a proper approach for improved control of both pathogens under field conditions.

## Conclusions

Results of the present study proved that the test vaccine Porcilis PCV M Hyo ID can be considered as safe to use on the 3rd week of age. The efficacy of the test vaccine was also highlighted on the specific trial farm with concurrent presence of M hyo and PCV2. Vaccination significantly reduced the PCV2 viraemia and shedding in faeces, whereas the severity of M hyo like lung lesions and pleuritis were also reduced in vaccinated animals. An improvement of ADWG during the finishing period and the overall study period was also observed. Taken together, based on the safety and efficacy results of the present study, the test vaccine can be suggested as a preventive tool under field conditions of concurrent exposure to M hyo and PCV2.

## Data Availability

The data presented in this study are available on reasonable request from the corresponding author after permission of the Funding Body.

## Abbreviations

BW: Body weight
ADWG: Average daily weight gain
PRDC: Porcine respiratory disease complex
PCV2: Porcine circovirus 2
M hyo: *Mycoplasma hyopneumoniae*
PCVD: Porcine circovirus diseases
PCV-2-SI: Porcine circovirus subclinical infection
PCV-2-SD: Porcine circovirus systemic disease
PCV-2-RD: Porcine circovirus reproductive disease
PDNS: Porcine dermatitis and nephropathy syndrome
IM: Intramuscular vaccination
ID: Intradermal vaccination
IDAL: Intradermal application of liquids
LI: *Lawsonia intracellularis*
APP: *Actinobacillus pleuropneumoniae*
HPS: *Hemophilus p*arasuis– *Glässerella parasuis*
PRRSV: Porcine Reproductive and Respiratory syndrome virus
LLS: Lung lesion scores
